# Health Information Obtained From the Internet and Changes in Medical Decision Making: Questionnaire Development and Cross-Sectional Survey

**DOI:** 10.2196/jmir.9370

**Published:** 2018-02-12

**Authors:** Yen-Yuan Chen, Chia-Ming Li, Jyh-Chong Liang, Chin-Chung Tsai

**Affiliations:** ^1^ Graduate Institute of Medical Education & Bioethics National Taiwan University College of Medicine Taipei Taiwan; ^2^ Department of Family Medicine National Taiwan University Hospital Bei-Hu Branch Taipei Taiwan; ^3^ Program of Learning Sciences National Taiwan Normal University Taipei Taiwan

**Keywords:** internet, help-seeking behavior, literacy, decision making

## Abstract

**Background:**

The increasing utilization of the internet has provided a better opportunity for people to search online for health information, which was not easily available to them in the past. Studies reported that searching on the internet for health information may potentially influence an individual’s decision making to change her health-seeking behaviors.

**Objective:**

The objectives of this study were to (1) develop and validate 2 questionnaires to estimate the strategies of problem-solving in medicine and utilization of online health information, (2) determine the association between searching online for health information and utilization of online health information, and (3) determine the association between online medical help-seeking and utilization of online health information.

**Methods:**

The Problem Solving in Medicine and Online Health Information Utilization questionnaires were developed and implemented in this study. We conducted confirmatory factor analysis to examine the structure of the factor loadings and intercorrelations for all the items and dimensions. We employed Pearson correlation coefficients for examining the correlations between each dimension of the Problem Solving in Medicine questionnaire and each dimension of the Online Health Information Utilization questionnaire. Furthermore, we conducted structure equation modeling for examining the possible linkage between each of the 6 dimensions of the Problem Solving in Medicine questionnaire and each of the 3 dimensions of the Online Health Information Utilization questionnaire.

**Results:**

A total of 457 patients participated in this study. Pearson correlation coefficients ranged from .12 to .41, all with statistical significance, implying that each dimension of the Problem Solving in Medicine questionnaire was significantly associated with each dimension of the Online Health Information Utilization questionnaire. Patients with the strategy of online health information search for solving medical problems positively predicted changes in medical decision making (*P*=.01), consulting with others (*P*<.001), and promoting self-efficacy on deliberating the online health information (*P*<.001) based on the online health information they obtained.

**Conclusions:**

Present health care professionals have a responsibility to acknowledge that patients’ medical decision making may be changed based on additional online health information. Health care professionals should assist patients’ medical decision making by initiating as much dialogue with patients as possible, providing credible and convincing health information to patients, and guiding patients where to look for accurate, comprehensive, and understandable online health information. By doing so, patients will avoid becoming overwhelmed with extraneous and often conflicting health information. Educational interventions to promote health information seekers’ ability to identify, locate, obtain, read, understand, evaluate, and effectively use online health information are highly encouraged.

## Introduction

Just several decades ago, people relied solely on traditional media to obtain health information, for example, reading newspaper and magazines, listening to radio, watching television, and seeking physicians’ advices. With the rapid advance of modern media technology, access to the internet has been dramatically growing. The increasing utilization of the internet has provided a better opportunity for people to search for health information online, which was not easily available to them in the past, regardless of its credibility, accuracy, and reliability.

According to a report published by the Taiwan Network Information Center in 2016, 18.81 million (89.39%) of people aged 12 years and older in Taiwan used the internet [1]. In addition, 14.48 (76.98%) and 15.41 (81.92%) million of them were mobile internet users and wireless internet users, respectively [2]. Not only Taiwan has its own search engines [3] in its written language—Traditional Chinese—but several popular global search engines, for example, Yahoo [4] and Google [5], also have a Traditional Chinese version [6,7]. People in Taiwan are free to use the internet without any censorship and control from Taiwan’s Government. Among those internet users, a certain number of them search online for health information. In the United States, based on a report published by the Pew Research Center, 87% of the total population used the internet, and the offline population has gradually declined from 48% of the total population in 2000 to 13% of the total population in 2016 [8]. According to a telephone survey conducted in 2010 by the Pew internet Project and California Health Care Foundation, approximately 80% of internet users in the United States searched online for health information. Among those internet users, 66% searched for health information about a disease or a medical problem, 56% searched for a medical treatment or a procedure, and 44% searched for information related to health care professionals [9]. Along with the advance of internet technology, searching online for health information is becoming more and more popular among patients and the general population.

Nevertheless, the credibility, accuracy, and reliability of health information on the internet are of great concern. Taking extracorporeal membrane oxygenation, a life-supporting treatment, as an example, a group of researchers reported that health information found on the internet, as indicated by the survival rate of extracorporeal membrane oxygenation users, was distorted as compared with that reported in the literature, and was much more distorted than health information found in the traditional media, for example, the newspaper [10]. This distorted online health information may potentially influence health information seekers’ attitude toward a disease, a medical treatment, or a procedure.

Whether health information reported on the internet is associated with the change of health-seeking behaviors has also been studied. Hsieh et al conducted a questionnaire study based on a random sample collected in Taiwan to examine the association between an individual’s internet usage and her ambulatory care utilization. They found that an individual with more frequent use of the internet was more likely to utilize more medical care as indicated by ambulatory care visits [11]. Furthermore, significant social events, particularly highlighted on the internet and traditional media, were very likely to encourage the use of life-supporting treatments [12]. Those studies implied that searching on the internet for health information may potentially influence an individual’s decision making to change her health-seeking behaviors.

Although distorted health information on the internet and its association with health-seeking behaviors have been studied, whether health information obtained online influences the medical decision making of the health information seekers is, to the best of our knowledge, under-researched. The objectives of this study were: (1) to develop and validate 2 questionnaires to estimate the strategies of problem solving in medicine and the online health information utilization; (2) to determine the association between searching online for health information and online health information utilization, and (3) to determine the association between online medical help-seeking and online health information utilization.

## Methods

### Settings and Participants

Participants of this study were recruited purposefully by inquiring with people in the waiting area of the outpatient clinic in a university-affiliated community teaching hospital located at Northern Taiwan from September 7, 2015, to December 31, 2015. The university-affiliated community teaching hospital has 31 acute care beds, with a total of approximately 1000 outpatient clinic visits per day on average. After the patients agreed to participate in this study, the paper-and-pencil format of the Problem Solving in Medicine (PSM) and Online Health Information Utilization (OHIU) questionnaires were handed to them, and then collected after they completed.

### Hypotheses

On the basis of the objectives, this study was set out to test the following 5 hypotheses:

- Hypothesis 1: Patients who prefer to search online health information for solving medical problems are more likely to change medical decisions based on the obtained information.
- Hypothesis 2: Patients who prefer to search online health information for solving medical problems are more likely to consult other people for further discussions based on the obtained information.
- Hypothesis 3: Patients who prefer to search online health information for solving medical problems are more likely to have high self-efficacy on deliberating the online health information.
- Hypothesis 4: Patients with the strategy of online formal medical help-seeking for solving medical problems predict how they handle online health information.
- Hypothesis 5: Patients with the strategy of online informal medical help-seeking for solving medical problems predict how they handle online health information.

### Instruments

To assess the association between patients’ strategies of problem solving in medicine and their online medical information utilization, 2 questionnaires were implemented in this study.

The PSM questionnaire was developed based on the online academic help-seeking questionnaire [13]. It consists of 3 dimensions—information searching, formal query, and informal query—which attempt to examine students’ online academic help-seeking when they access academic information on the internet. The three-dimensional structure was utilized for the development of the PSM questionnaire and was specially designed for solving medical problems in this study.

To construct a valid and reliable questionnaire, more dimensions were added, including items designed specifically for the participants. In total, 2 faculty members specializing in education and 1 senior attending physician working in a university-affiliated community hospital validated the items for 2 rounds. In the first round, the 3 dimensions in paper-and-pencil format were reviewed, and the “nononline version” framework for each dimension was suggested. In the second round, some revisions for the wording of the questionnaire items were suggested during a group discussion.

A total of 27 items were included in the PSM questionnaire, and these items belonged to the following 6 dimensions: nononline health information search (NHIS), online health information search (OHIS); nononline formal medical help-seeking (NFMH), online formal medical help-seeking (OFMH), nononline informal medical help-seeking (NIMH), and online informal medical help-seeking (OIMH). Each dimension of the questionnaire contained a total of 4 to 5 items. The PSM questionnaire used a Likert scale ranging from 1 to 5, indicating a participant’s agreement with each item from *strongly disagree* to *strongly agree*, respectively. The details of the 6 dimensions are as follows (see Multimedia Appendix 1):

1. NHIS: assessing the extent to which participants seek related nononline health information to solve their medical problems (eg, searching medical textbooks, magazines, or newspapers).
  Sample item: “When I have a medical problem, I will search for relevant knowledge from professional medical books.”
2. OHIS: assessing the extent to which participants seek related online health information to solve their medical problems (eg, searching the internet via search engines). Sample item: “When I have a medical problem, I will search for solutions using internet search engines (eg, Google, Yahoo).”
3. NFMH: assessing the extent to which participants query medical experts about solving medical issues (eg, consulting the physician in the hospital directly). Sample item: “When I don’t know how to solve a medical problem, I will call a doctor, a medical expert, or other health care professionals for help.”
4. OFMH: assessing the extent to which participants query medical experts through the internet about solving medical issues (eg, consulting the physician by email or through the Web). Sample item: “When I can’t solve a medical problem, I will email a doctor, a medical expert, or a health care professional to seek medical helps.”
5. NIMH: assessing the extent to which participants seek medical helps from their family members or experienced people (eg, querying family members about previous medical problem-solving experiences). Sample item: “When I have a medical problem, I will seek helps from a drugstore.”
6. OIMH: assessing the extent to which participants seek medical helps from unknown experts on the internet (eg, posting medical help requests on Web forums). Sample item: “When I have a medical problem, I will post a message on relevant Web forums requesting help from other forum users.”

The second questionnaire, the OHIU questionnaire, was specifically developed for the participants in this study. The dimensions and items of the OHIU questionnaire were developed based on face-to-face interviews with 16 patients (8 males and 8 females, with an average age of 45 years), who were recruited at a university-affiliated community hospital. The researchers invited the patients who claimed they had experiences of and showed interest in searching online for health information to participate in a face-to-face interview. A semistructured interview about his/her OHIU was conducted based on the following interview questions: (1) why did you search for online health information? (2) what was your attitude toward the online health information you obtained? and (3) what did you do after searching for the online health information?

The 2 faculty members and the senior attending physician who helped validate the PSM questionnaire also validated the dimensions and items of the OHIU questionnaire. The OHIU finally included 3 dimensions: “changing decisions” (CD), “consulting others” (CO), and “promoting self-efficacy” (PS). Each dimension has a total of 4 items. The OHIU questionnaire used a Likert scale ranging from 1 to 5, indicating a participant’s agreement to each item from *strongly disagree* to *strongly agree*, respectively. The details of the 3 dimensions are as follows (Multimedia Appendix 2):

1. CD: assessing the extent to which participants change medical decisions based on the online health information they obtained. Sample item: “After searching for online health information, I will change my views to align with the information I obtained.”
2. CO: assessing the extent to which participants consult other people based on the online health information they obtained. Sample item: “Online health information is an important reference for me when making medical decisions.”
3. PS: assessing the extent to which participants promote self-efficacy in deliberating the online health information based on the online health information they obtained. Sample item: “I am confident that I can evaluate the accuracy of online health information for making medical decisions.”

### Data Analysis

We conducted confirmatory factor analysis (CFA) to test the hypotheses about the structure of the factor loadings and intercorrelations for all the items and dimensions of the PSM and OHIU questionnaires. To ensure the convergent validity of this proposed measurement model, 3 rules were followed: (1) all of the items in the CFA standardized factor loadings should be higher than .60; (2) the values of the composite reliability should exceed .80; and (3) the average variance extracted should exceed .50 [14-17]. The items that did not fulfill the 3 rules were excluded from further analysis.

For examining the correlations between each dimension of the PSM questionnaire and each dimension of the OHIU questionnaire, we employed Pearson correlation coefficients.

Furthermore, we conducted structure equation modeling for examining the possible linkage between each of the 6 dimensions of the PSM questionnaire and each of the 3 dimensions of the OHIU questionnaire. We examined model fit using squared multiple correlation (*R*^2^).

All the statistical analyses were conducted using IBM SPSS AMOS 20. A *P* value of less than .05 was regarded as statistically significant. This study was approved by the Hospital Committee. All procedures performed in studies involving human participants were in accordance with the ethical standards of conducting a questionnaire study. Informed consent was obtained from all individual participants included in the study.

## Results

### Participants

A total of 457 patients (258 females and 199 males) participated in this study. The age of the participating patients ranged from 14 to 79 years, with a mean of 44.77 (SD 11.87) years. Of the 457 participants, 1.1% (5/457) had an educational level of elementary school or lower, 3.5% (16/457) had a level of junior high school, 20.4% (93/457) had a level of senior high school, 60.4% (276/457) had college or university degrees, and 14% (64/457) had graduate degrees or above. Moreover, 76.2% (348/457) of the patients indicated that their weekly time for internet usage was approximately 10 hours. Most of the patients (99.1%, 453/457) reported that they searched online for health information from *occasionally* to *always*.

### Structure of Measurement

It has been suggested that the measurement model for each dimension in the structural model is estimated first in the process of examining the structural model, and then the final whole structural model is tested [18]. Regarding examining the measurement model, a single CFA with all the dimensions and its items of the PSM and OHIU questionnaires included in one model was conducted to clarify the model structure, validity, and reliability of both questionnaires. Multimedia Appendix 3 shows the CFA results.

A total of 31 items, 21 for the PSM questionnaire and 10 for the OHIU questionnaire, were kept in the final measurement model. All of the standardized factor loadings for this measurement model were close to or above .60 (between .56 and .96) and statistically significant (*t* statistics ranged from 11.33 to 22.90), supporting the sufficient factors in this model. All of the composite reliability coefficients exceeded .80 (between .81 and .91). All of the average variance extracted values exceeded .50 (between .55 and .77). Cronbach alpha values ranged from .79 to .90. The above validity values showed an adequate representation of convergent and construct validity for this proposed model. With respect to the goodness-of-fit indices of this model for the 2 questionnaires, the following data were obtained: Chi-square=1280.3, degree of freedom=398, the ratio of Chi-square to degrees of freedom=3.22, the standardized root mean square residual=0.065, the root mean square error of approximation=0.070, the goodness-of-fit index=0.84, the adjusted goodness-of-fit index=0.80, the incremental fit index=0.90, and the comparative fit index=0.90. The results of the analysis showed perfect or acceptable fitness values that supported the acceptable model fit for both questionnaires in this model.

### The Relationships Between the Problem Solving in Medicine and Online Health Information Utilization Questionnaires

We conducted Pearson correlation coefficients to examine the relationships between the 6 dimensions of the PSM questionnaire and the 3 dimensions of the OHIU questionnaire (Table 1; Multimedia Appendix 4). We found that the linear relationships between each of the 6 dimensions in the PSM questionnaire and each of the 3 dimensions in the OHIU questionnaire, although they only ranged from weak (*r*=.12) to moderate (*r*=.41), were significantly different from 0—no linear relationship (*P*<.05). The results implied that each dimension of the PSM questionnaire was significantly associated with each dimension of the OHIU questionnaire. That is, all of the proposed hypotheses were positively supported based on Pearson correlation coefficients.

### Test of the Structural Model

**Figure 1:**
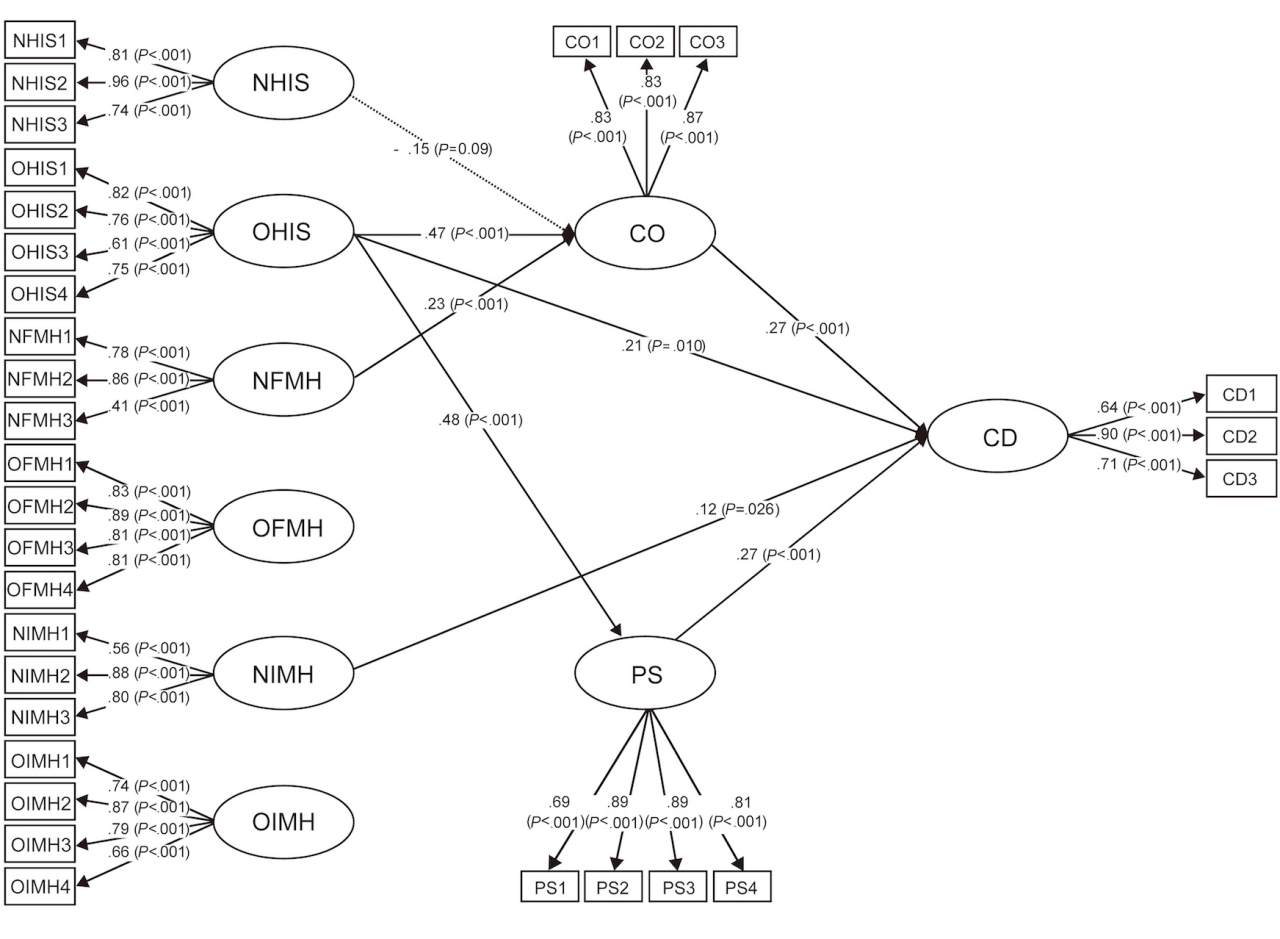
The structural equation model of the relationships between patients’ responses to Problem Solving in Medicine and Online Health Information Utilization questionnaires. CD: changing decisions; CO: consulting others; NFMH: nononline formal medical help-seeking; NHIS: nononline health information search; NIMH: nononline informal medical help-seeking; OFMH: online formal medical help-seeking; OHIS: online health information search; OIMH: online informal medical help-seeking; PS: promoting self-efficacy.

Based on the correlation results, a structural equation model was further conducted to analyze the structural path relationships among patients’ strategies of problem solving in medicine and online health information utilization. Figure 1 shows the structural relationships by means of path coefficients among the dimensions of the 2 questionnaires. The standardized and statistical significant path coefficients were indicated by *P* values. In addition, the path with no statistical significance in this model is omitted. The results indicated that the test of the structural model showed a good or acceptable model fit (Chi-square=1380.05, degree of freedom=399, the ratio of Chi-square to degrees of freedom=3.46, the standardized root mean square residual=0.077, the root mean square error of approximation=0.073, the goodness-of-fit index=0.83, the adjusted goodness-of-fit index=0.79, the incremental fit index=0.88, and the comparative fit index=0.88). These indices revealed that this structural model sufficiently explained the collected data.

**Table 1:** The correlations among the dimensions of the Problem Solving in Medicine and Online Health Information Utilization questionnaires (Pearson correlation coefficient and *P* value).

Problem Solving in Medicine questionnaire	Online Health Information Utilization questionnaire					
	NHIS^a^	OHIS^b^	NFMH^c^	OFMH^d^	NIMH^e^	OIMH^f^
Changing decisions	.20 (*P*<.001)	.40 (*P*<.001)	.18 (*P*<.001)	.23 (*P*<.001)	.23 (*P*<.001)	.25 (*P*<.001)
Consulting others	.18 (*P*<.001)	.41 (*P*<.001)	.33 (*P*<.001)	.25 (*P*<.001)	.14 (*P*=.004)	.24 (*P*<.001)
Promoting self-efficacy	.25 (*P*<.001)	.41 (*P*<.001)	.24 (*P*<.001)	.28 (*P*<.001)	.12 (*P*=.008)	.25 (*P*<.001)

^a^NHIS: nononline health information search.

^b^OHIS: online health information search.

^c^NFMH: nononline formal medical help-seeking.

^d^OFMH: online formal medical help-seeking.

^e^NIMH: nononline informal medical help-seeking.

^f^OIMH: online informal medical help-seeking.

As shown in Figure 1, patients with the strategy of OHIS for solving medical problems positively predicted CD (Hypothesis 1; path coefficient=.21; *P*=.01), CO (Hypothesis 2; path coefficient=.47; *P*<.001), and PS on deliberating the online health information (Hypothesis 3; path coefficient=.48; *P*<.001) based on the online health information they had obtained. In comparison, patients’ strategy of OFMH when encountering medical problems was not significantly associated with any dimensions of their OHIU (Hypothesis 4), and OIMH was not significantly associated with any dimensions of their OHIU (Hypothesis 5).

The squared multiple correlations (*R*^2^) of the 3 dependent variables (CD, CO, and PS) ranged from .29 to .38 (CD: .38; CO: .31; PS: .29), implying that each of the dependent variables was explained by its predictors at a range from 29% (PS) to 31% (CO). In addition, all of the independent variables in this study (6 dimensions in the problem solving in medicine questionnaire; PS and CO in the online health information utilizationquestionnaire) explained 38% of the total variance in participants’ change of medical decision making (CD).

## Discussion

### Principal Findings

Our study found that patients’ strategies of problem solving in medicine were associated with their online health information utilization. In particular, patients who engaged in online health information search for solving medical problems were significantly and positively associated with changing their medical decisions. Therefore, the more online health information patients searched for solving their medical problems, the more likely their medical decision was changed based on the online health information they had gathered.

Patients using the strategy of nononline formal medical help-seeking when encountering medical problems were significantly associated with consultation with others about the online health information they obtained. Furthermore, those using the strategy of nononline informal medical help-seeking were significantly associated with changing medical decisions based on the online health information. In comparison, patients with the strategy of nononline health information searches, for example, searching medical textbooks, medical magazines, and newspapers/TV news, were significantly and negatively associated with consulting others based on the online health information they had gathered.

### Online Health Information and Decision Making

As Lagan and colleagues reported based on a -based study, 83% of the pregnant women changed their decisions based on the online health information they had searched for and obtained [19]. Bert and colleagues also found that pregnant women were more likely to change their lifestyles if the searched online health information included the lifestyle topics of nutrition, physical activities, and alcohol consumption during pregnancy [20]. Our study results showing the association between online health information searches and medical decision changes were echoed by several other studies conducted using different study designs.

On the basis of the Porter Novelli Health Styles database, Dutta-Bergman examined online health information seekers’ health attitude, health cognitions, and health behaviors. Dutta-Bergman reported that individuals searching for health information on the internet were more likely to have better health information orientation, health-oriented beliefs, and healthy activities than those who did not [21]. Certainly, such individuals undoubtedly may easily change their decisions if they consider that their current health-related conditions are not consistent with the information they have obtained from the internet.

Our study results also highlighted that the credibility of online health information plays a very important role in medical decision making. Although most health information seekers are usually skeptical of trusting health information reported on the internet, they may not pay attention to how they select the online health information. For example, a qualitative study concluded that, although online health information seekers recognized the online health information from governments, organizations, and educational institutions as credible, they usually selected the health information from the first page of the search results by looking for keywords or short descriptions using search engines such as Google and Yahoo [22]. However, the adjusted odds that a patient changed her decision after searching for the online health information from health care institutions increased by 79% as compared with the adjusted odds after searching for the online health information not from health care institutions [20]. These results imply that both the online health information providers and the online health information seekers certainly play a major role in evidence-based medical decision making.

Online health information providers may benefit evidence-based medical decision making by providing online health information seekers with valid, accurate, and credible health information. However, health information reported in the media is commonly distorted [23,24], and the distortion of health information on the internet is still commonplace [10,12]. Given that online health information seekers recognize online health information from governments, organizations, and educational institutions as more credible than that from other websites [22], it is suggested that governments, professional organizations, educational institutions, and any other recognized online health information providers take responsibility for providing valid, accurate, and credible online health information.

On the other hand, online health information seekers not only receive the health information on the internet but also synthesize, disseminate, and exchange that information [25]. Therefore, online health information seekers’ ability to identify, locate, obtain, read, understand, evaluate, and effectively use online health information (health literacy, information literacy, and media literacy) is of great importance to make evidence-based medical decisions. Any educational interventions that may promote online health information seekers’ ability to receive, synthesize, disseminate, and exchange online health information are highly encouraged.

Although searching online for health information predicted change of medical decision making in this study, only 38% of the total variance for the dependent variable, CD, was accounted for in this model, leaving 62% unexplained. Other potential variables to explain the variance of change of medical decision making can be further investigated in the future studies.

### Online Health Information Management

In addition to changing medical decisions, online health information seekers, when solving medical problems, may also use online health information to supplement their consultations with others, for example, physicians, family members, and experts. One reason that a large proportion (94%) of pregnant women searched online for health information was to verify the health information already given by health care professionals [19]. Similar findings concluding that patients use online health information to validate health information gathered from health care professionals were also reported by a qualitative study based on 13 asynchronous online focus groups from 5 countries [26]. Our study showed that patients with the strategy of searching online for health information when solving medical problems tended to validate the online health information by consulting with others. Accordingly, the validation of information is bidirectional by either validating health information from health care professionals by searching the internet or validating health information on the internet by consulting with other people.

We also reported that patients using the strategy of online health information search in the face of medical problems were associated with having high self-efficacy on: (1) searching for valid, accurate, and credible health information; (2) using the health information; and (3) making correct judgments. Our study result was similar to that reported by Bert and colleagues, pointing out that pregnant women had more confidence in health information after they had verified the information through internet searches [20], and also supported by Pang and colleagues’ report, suggesting that people will look for different sources to validate and evaluate health information [27].

### Nononline Medical Help-Seeking

Patients with the strategy of nononline formal medical help-seeking when encountering medical problems tended to consider online health information as a supplement to their further consultation with others. Studies have shown that the patients with greater intention to seek medical help from professional resources, for example, physicians, are more likely to have higher levels of health literacy [28], and patients seeking online formal medical help are more likely to have higher levels of eHealth literacy [29]. Suka and colleagues also reported that health literacy was positively associated with help-seeking intentions, and a majority of the study participants chose to seek help at any time from formal resources [30]. Given that our study patients, preferring to seek help from formal sources, might have better health literacy, consulting with others for further verifying the online health information should be expected.

In comparison, those with the strategy of nononline informal medical help-seeking—for example, seeking help from pharmacist, family members, and friends with experiences, and by praying to god for advice—were more likely to change their medical decisions based on the online health information they obtained. Kleinman and colleagues proposed that family members, friends, other community leaders, and folk healers have played and still play an important role on how people perceive and solve medical problems [31]. Griffiths and colleagues reported that informal help from friends and family members has advantages, such as social, emotional, informational, and companionship support, as well as disadvantages, namely, stigma and inappropriate support due to friends’ or family members’ lack of related knowledge [32]. Changing medical decisions following the suggestions given by those with insufficient knowledge can be unhelpful or even toxic. Therefore, providing educational interventions for promoting nononline informal medical help-seekers’ ability to obtain, read, understand, evaluate, and use health information is highly suggested.

### Nononline Health Information Search

This study also found that nononline health information search, for example, searching medical textbooks, medical magazines, and newspapers/TV news, was significantly and negatively associated with consulting with others based on the online health information. With the increasing and prevalent use of the internet as a tool for medical help-seeking, those who still prefer to seek medical help through nononline ways may have negative attitude toward trusting online health information, and thus are less likely to consult with others based on the online health information they have already recognized as doubtful. The negative attitude toward online health information is reflected on the statistically insignificant associations between the strategy of nononline health information search and changing medical decisions and between the strategy of nononline health information search and having high self-efficacy on searching for valid and credible health information, using the health information, and making correct judgments.

Although NHIS was positively associated with CO (*r*=.18, *P*<.001) in Pearson correlation coefficients, NHIS became a negative predictor in the structural equation model. This negative suppression effect may occur when both the independent variables positively and highly correlate to each other, with a positive zero-order correlation to the dependent variable [33,34]. A previous study suggested to delete one of the two independent variables or to combine the two independent variables into a single variable [35].

In this study, we found that NHIS and OHIS were positively and highly associated with each other (*r*=.47, *P*<.001). However, we did not delete either NHIS or OHIS, nor did we combine the two independent variables into a single variable because the two independent variables were of great importance and also the focuses in this study. As a result, the negative suppression effect occurred, and NHIS became a negative predictor of CO.

### Strengths and Limitations

Few studies have been conducted for examining the associations between the strategies of problems solving in medicine and online health information utilization, with a particular focus on the association between online health information search and changing medical decisions. Moreover, the large number of patients recruited for this study strengthens the extrapolation of the study results. Nevertheless, some limitations are inevitable in this study.

The first limitation is attributable to the sampling method used in this study. The participants were purposefully recruited in the outpatient clinics of a university-affiliated community hospital located at Northern Taiwan. The generalizability of the results of a study conducted by purposeful sampling in a single center rather than different settings might be of concern. In addition, participants were recruited from the waiting area of the outpatient clinics. They might be more likely to select nononline formal medical health-seeking as their strategies and their health condition might be worse than that of the general population. Both of these issues might further limit the generalizability of the study results.

Considerable factors, such as age, gender, and educational level, surround medical decision making. However, some of those factors surrounding medical decision making were not controlled in this study. For example, severity of illness may influence a patient’s medical decision making, but it was not collected and controlled in this study.

This was an attitudinal study conducted using valid and reliable questionnaires to measure the relationships between the strategies of problems solving in medicine and online health information utilization. However, this was not a field study in which the influence of online health information search on changing medical decisions can be directly observed and measured. Whether an association identified in a questionnaire study reflects exactly the same association in a field study is not guaranteed.

As some of the participants were outpatient clinic patients, they might have chronic diseases and have relatively higher health literacy regarding their chronic problems. Their perceptions of the online health information may be different from new patients. However, we did not adjust for their chronic diseases status.

### Conclusions

With the growing use of the internet and the dramatic proliferation of health information on the internet, the findings from this study suggest that the influence that online health information has on online health information seekers’ medical decision making is visible. Therefore, the credibility of online health information plays a very important role on medical decision making. Present health care professionals have a responsibility to acknowledge that, regardless of the credibility of online health information, patients’ medical decision making may be changed following the health information reported on the internet. Health care professionals should assist patients’ medical decision making by initiating as much dialogue with patients as possible, providing credible and convincing health information to patients, and guiding patients where to look for accurate, comprehensive, and understandable online health information. By doing so, patients will avoid becoming overwhelmed with extraneous and often conflicting health information. Educational interventions to promote health information seekers’ ability to identify, locate, obtain, read, understand, evaluate, and effectively use online health information are highly encouraged.

## Appendix

Multimedia Appendix 1 The Problem Solving in Medicine questionnaire.

Multimedia Appendix 2 The Online Health Information Utilization questionnaire.

Multimedia Appendix 3 The confirmatory factor analysis for the Problem Solving in Medicine and Online Health Information Utilization questionnaires (N=457).

Multimedia Appendix 4 The scatter plot of each of the 6 dimensions in the Problem Solving in Medicine questionnaire and each of the 3 dimensions in the Online Health Information Utilization questionnaire.

## Abbreviations

CD: changing decisions
CFA: confirmatory factor analysis
CO: consulting others
NFMH: nononline formal medical help-seeking
NHIS: nononline health information search
NIMH: nononline informal medical help-seeking
OFMH: online formal medical help-seeking
OHIS: online health information search
OHIU: Online Health Information Utilization questionnaire
OIMH: online informal medical help-seeking
PS: promoting self-efficacy
PSM: Problem Solving in Medicine questionnaire
