# Supplementary material for: Health Information Obtained From the Internet and Changes in Medical Decision Making: Questionnaire Development and Cross-Sectional Survey
Source: J Med Internet Res. 2018 Feb 12;20(2):e47. doi: 10.2196/jmir.9370 (PMC5826978; doi:10.2196/jmir.9370)
Supplement: Multimedia Appendix 1 [file jmir_v20i2e47_app1.pdf]

Appendix 1. The Problem-solving in Medicine questionnaire (PSM).

		Items
**NHIS 1**	1	When I have a medical problem, I will search for relevant knowledge from professional medical books.
**NHIS 2**	2	When I have a medical problem, I will search for relevant knowledge from medical magazines.
**NHIS 3**	3	When I have a medical problem, I will search for relevant knowledge from news reporting.
**NHIS 4**	4	When I have a medical problem, I will search for relevant knowledge from TV or radio programs.
**OHIS 1**	5	When I have a medical problem, I will search for solutions using internet search engines (e.g., Google, Yahoo).
**OHIS 2**	6	When I have a medical problem, I will search for solutions from online discussion forums (e.g., medical-related online forum, bulletin board system (BBS)).
**OHIS 3**	7	When I have a medical problem, I will search for solutions from the websites of the social media platforms and social networking services (e.g., Facebook, Twitter).
**OHIS 4**	8	When I have a medical problem, I will search for solutions from medical websites (hospital websites, hospital e-newspapers).
**OHIS 5**	9	When I have a medical problem, I will search for solutions from doctors' websites.
**NFMH 1**	10	When I don't know how to solve a medical problem, I will call a doctor, a medical expert, or other health care professionals for helps.
**NFMH 2**	11	When I have a medical problem, I will seek helps from doctors or other health care professionals who are known by my friends or relatives.
**NFMH 3**	12	When I have a medical problem, I will seek helps from doctors or health care professionals who are working in health care institutions.
**NFMH 4**	13	When I have a medical problem, I will inquire the pharmacist of a nearby pharmacy store.
**OFMH 1**	14	When I can't solve a medical problem, I will e-mail a doctor, medical expert or health care professional to seek medical help.
**OFMH 2**	15	When I have a medical problem that I don't understand, I will post a request on internet medical forums for help from doctors, other health care professionals, or medical experts.
**OFMH 3**	16	When I have a medical problem, I will seek help from doctors or other health care professionals using Instant Messages (e.g.,

		Facebook, Line, WeChat, WhatsApp).
**OFMH 4**	17	When I have a medical problem, I will try to seek help from websites or online blogs maintained by doctors or health care institutions.
**NIMH 1**	18	When I have a medical problem, I will seek helps from a drugstore.
**NIMH 2**	19	When I have a medical problem, I will ask for advice or opinions from my family members.
**NIMH 3**	20	When I have a medical problem, I will go to the temple praying to god for advice.
**NIMH 4**	21	When I have a medical problem, I will seek help from experienced persons.
**NIMH 5**	22	When I have a medical problem, I will seek help from friends or relatives.
**OIMH 1**	23	When I have a medical problem, I will post a message on relevant web forums requesting help from other forum users.
**OIMH 2**	24	When I can’t solve a medical problem, I will seek help from others using my microblog (e.g., Facebook or Twitter).
**OIMH 3**	25	When I have a medical problem, I will seek help from friends, colleagues or netizens using Instant Messages (e.g., Facebook, Line, WeChat, WhatsApp).
**OIMH 4**	26	When I have a medical problem, I will post the problem on relevant websites of knowledge communities (e.g., Facebook) and try to find a solution.
**OIMH 5**	27	When I have a medical problem, I will post the problem on a proper website, medical-related online forums or bulletin board system (BBS).

Abbreviation List: NHIS = non-online health information search; OHIS = online health information search; NFMH = non-online formal medical help-seeking; OFMH = online formal medical help-seeking; NIMH = non-online informal medical help-seeking; OIMH = online informal medical help-seeking.
