# Supplementary material for: Health Information Obtained From the Internet and Changes in Medical Decision Making: Questionnaire Development and Cross-Sectional Survey
Source: J Med Internet Res. 2018 Feb 12;20(2):e47. doi: 10.2196/jmir.9370 (PMC5826978; doi:10.2196/jmir.9370)
Supplement: Multimedia Appendix 2 [file jmir_v20i2e47_app2.pdf]

Appendix 2. The Online Health Information Utilization questionnaire (OHIU).

		**Items**
**CD 1**	1	After searching for online health information, I will change my views to align with the information I obtained.
**CD 2**	2	After searching for online health information, I will change my decision, aligning with the information I obtained, about whether to see a doctor.
**CD 3**	3	After searching for online health information, I will change my judgments, aligning with the information I obtained, on personal medical issues.
**CD 4**	4	After searching for online health information, I will change my decision, aligning with the information I obtained, about whether to consult an expert.
**CO 1**	5	Online health information is an important reference for me when making medical decisions.
**CO 2**	6	I will discuss relevant issues with a doctor based on the health information on the Internet.
**CO 3**	7	I will discuss relevant issues with my family or friends based on the health information on the Internet.
**CO 4**	8	I will discuss relevant issues with experts based on the health information on the Internet.
**PS 1**	9	I am confident that I can evaluate the accuracy of online health information for making medical decisions.
**PS 2**	10	I am confident that I can make good use of online medical information.
**PS 3**	11	I am confident that I can find useful online health information.
**PS 4**	12	I am confident that I can make correct medical judgments on personal medical issues based on online medical information.

Abbreviation List: CD = changing decisions; CO = consulting others; PS = promoting self-efficacy.
