# Supplementary material for: Health Information Obtained From the Internet and Changes in Medical Decision Making: Questionnaire Development and Cross-Sectional Survey
Source: J Med Internet Res. 2018 Feb 12;20(2):e47. doi: 10.2196/jmir.9370 (PMC5826978; doi:10.2196/jmir.9370)
Supplement: Multimedia Appendix 3 [file jmir_v20i2e47_app3.pdf]

Appendix 3. The CFA analysis for the Problem-Solving in Medicine and Online Information Utilization questionnaires. (N= 457)

Construct and Measurement Items	Factor Loading	t Statistics (*p* Value)	CR	AVE	Alpha Value
**The Problem Solving in Medicine questionnaire**					
**Non-online Health Information Search (NHIS) Mean = 3.26, SD = 0.42**	------	------	0.88	0.71	0.87
NHIS 1 (1)	0.81	------	------	------	------
NHIS 2 (2)	0.96	21.81 $p <.01$	------	------	------
NHIS 3 (3)	0.74	17.65 $p <.01$	------	------	------
**Online Health Information Search (OHIS) Mean = 3.49, SD = 0.77**	------	------	0.83	0.55	0.82
OHIS 1 (5)	0.82	------	------	------	------
OHIS 2 (6)	0.77	17.02 $p <.01$	------	------	------
OHIS 3 (7)	0.61	12.88 $p <.01$	------	------	------
OHIS 4 (9)	0.75	16.42 $p <.01$	------	------	------
**Non-online Formal Medical Help-seeking (NFMH) Mean = 3.41, SD = 0.84**	------	------	0.81	0.59	0.79
NFMH 1 (10)	0.75	------	------	------	------

NFMH 2 (11)	0.91	15.69 *p* <.01	------	------	------
NFMH 3 (12)	0.62	12.52 *p* <.01	------	------	------
**Online Formal Medical Help-seeking (OFMH)** **Mean = 2.65, SD = 0.87**	------	------	0.90	0.69	0.90
OFMH 1 (14)	0.83	------	------	------	------
OFMH 2 (15)	0.89	22.90 *p* <.01	------	------	------
OFMH 3 (16)	0.80	19.85 *p* <.01	------	------	------
OFMH 4 (17)	0.81	20.12 *p* <.01	------	------	------
**Non-online Informal Medical Help-seeking (NIMH)** **Mean = 3.46, SD = 0.77**	------	------	0.80	0.58	0.78
NIMH 1 (19)	0.56	------	------	------	------
NIMH 2 (21)	0.88	11.33 *p* <.01	------	------	------
NIMH 3 (22)	0.80	11.45 *p* <.01	------	------	------
**Online Informal Medical Help-seeking (OIMH)** **Mean = 2.62, SD = 0.83**	------	------	0.85	0.60	0.85
OIMH 1 (23)	0.74	------	------	------	------
OIMH 2 (24)	0.88	17.73	------	------	------

		p <.01			
OIMH 3 (25)	0.79	16.29 *p* <.01	------	------	------
OIMH 4 (27)	0.66	13.51 *p* <.01	------	------	------
**The Online Health Information Utilization questionnaire**					
**Changing Decisions (CD)**<br>**Mean = 3.25, SD = 0.70**	------	------	0.80	0.58	0.79
CD 1 (1)	0.64	------	------	------	------
CD 2 (2)	0.91	13.63 *p* <.01	------	------	------
CD 3 (3)	0.71	12.66 *p* <.01	------	------	------
**Consulting Others (CO)**<br>**Mean = 3.32, SD = 0.82**	------	------	0.91	0.77	0.88
CO 1 (6)	0.82	------	------	------	------
CO 2 (7)	0.95	20.36 *p* <.01	------	------	------
CO 3 (8)	0.86	20.77 *p* <.01	------	------	------
**Promoting Self-efficacy (PS)**<br>**Mean = 3.02, SD = 0.78**	------	------	0.89	0.68	0.89
PS 1 (9)	0.70	------	------	------	------
PS 2 (10)	0.89	17.51	------	------	------

		p <.01			
PS 3 (11)	0.89	17.46 *p* <.01	------	------	------
PS 4 (12)	0.80	16.00 *p* <.01	------	------	------

Abbreviation List: CFA = confirmatory factor analysis; CR = composite reliability; AVE = average variance extracted; SD = standard deviation; NHIS = Non-online Health Information Search; OHIS = Online Health Information Search; NFMH = Non-online Formal Medical Help-seeking; OFMH = Online Formal Medical Help-seeking; NIMH = Non-online Informal Medical Help-seeking; OIMH = Online Informal Medical Help-seeking; CD = changing decisions; CO = consulting others; PS = promoting self-efficacy.
