# Supplementary material for: Health Information Obtained From the Internet and Changes in Medical Decision Making: Questionnaire Development and Cross-Sectional Survey
Source: J Med Internet Res. 2018 Feb 12;20(2):e47. doi: 10.2196/jmir.9370 (PMC5826978; doi:10.2196/jmir.9370)
Supplement: Multimedia Appendix 4 [file jmir_v20i2e47_app4.pdf]

Appendix 4. The scatter plot of each of the six dimensions in the Problem-solving in Medicine questionnaire and each of the six dimensions in the Online Health Information Utilization questionnaire.

	NHIS	OHIS	NFMH	OFMH	NIMH	OIMH
CD	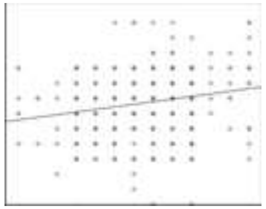	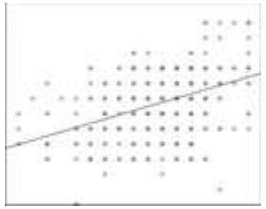	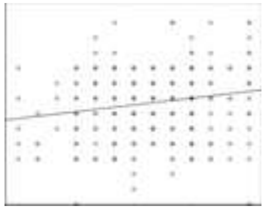	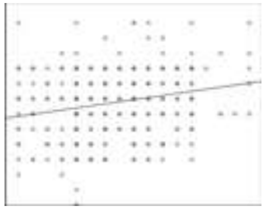	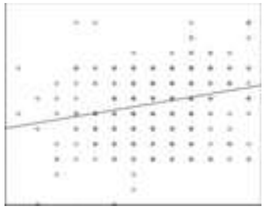	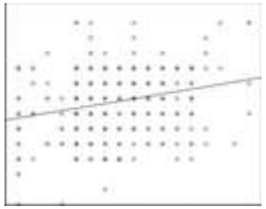
CO	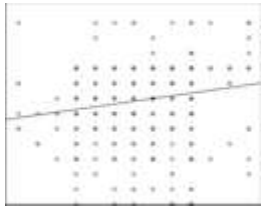	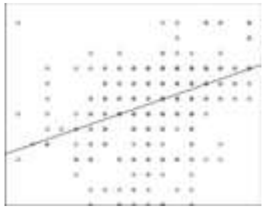	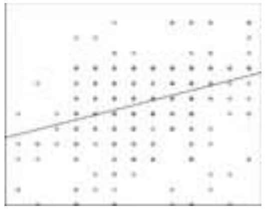	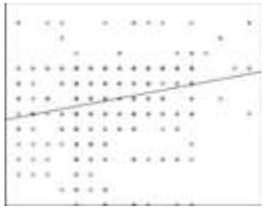	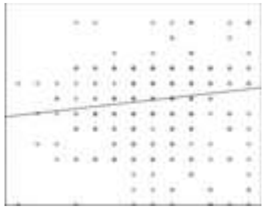	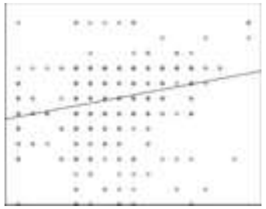
PS	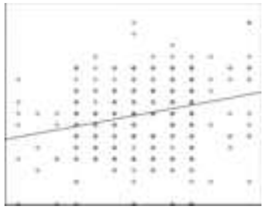	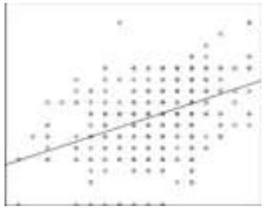	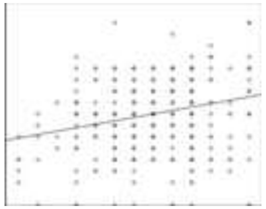	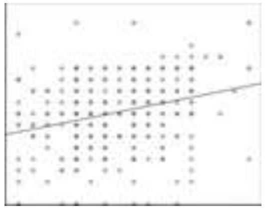	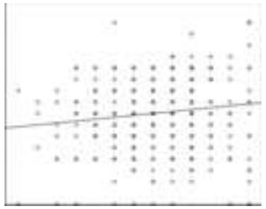	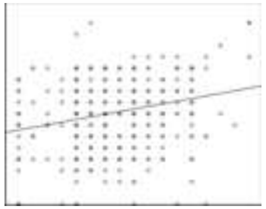

Abbreviation List: CD = changing decisions; CO = consulting others; PS = promoting self-efficacy; NHIS = Non-online Health Information

Search; OHIS = Online Health Information Search; NFMH = Non-online Formal Medical Help-seeking; OFMH = Online Formal Medical Help-seeking; NIMH = Non-online Informal Medical Help-seeking; OIMH = Online Informal Medical Help-seeking.
